# Integrative analysis of single-nucleus RNA sequencing and Mendelian randomization to explore novel risk genes for Alzheimer’s disease

**DOI:** 10.1097/MD.0000000000040551

**Published:** 2024-11-15

**Authors:** Chao Huang, Ruihao Zhou, Xingya Huang, Fanshu Dai, Biao Zhang

**Affiliations:** a Clinical College of Neurology, Neurosurgery and Neurorehabilitation, Tianjin Medical University, Tianjin, China; b Tianjin Key Laboratory of Cerebral Vascular and Neurodegenerative Diseases, Tianjin Neurosurgical Institute, Tianjin Huanhu Hospital, Tianjin, China.

**Keywords:** Alzheimer’s disease, Mendelian randomization, risk gene, single-nucleus RNA sequencing

## Abstract

In this study, we aimed to delineate cellular heterogeneity in Alzheimer’s disease (AD) and identify genetic markers contributing to its pathogenesis using integrative analysis of single-nucleus RNA sequencing (sn-RNA-Seq) and Mendelian randomization (MR). The dorsolateral prefrontal cortex sn-RNA-Seq dataset (GSE243292) was sourced from the Gene Expression Omnibus (GEO) database. Data preprocessing was conducted using the Seurat R software package, employing principal component analysis (PCA) and uniform manifold approximation and projection (UMAP) for cell clustering and annotation. MR analysis was used to identify instrumental variables from expression quantitative trait loci (eQTL) and GWAS data by applying inverse variance weighting (IVW), weighted median (WM) and MR-Egger methods. This was complemented by leave-one-out sensitivity analysis to validate the causal relationship on AD risk genes. We identified 23 distinct cell clusters, which were annotated into eight subgroups, including oligodendrocytes, oligodendrocyte precursors, astrocytes, macrophage cells, endothelial cells, glutamatergic neurons, neural stem cells, and neurons. Notably, the number of macrophages significantly increased in the AD group. Using genome-wide association study (GWAS) summaries and eQTL data, MR analysis identified causal relationships for 7 genes with significant impacts on AD risk. Among these genes, CACNA2D3, INPP5D, RBM47, and TBXAS1 were associated with a decreased risk of AD, whereas EPB41L2, MYO1F, and SSH2 were associated with an increased risk. A leave-one-out sensitivity analysis confirmed the robustness of these findings. Expression analysis revealed that these genes were variably expressed across different cell subgroups. Except for the CACNA2D3 gene, the other 6 genes showed increased expression levels in the macrophages, particularly EPB41L2 and SSH2. Our findings highlight the potential of specific genetic markers identified through integrative analysis of sn-RNA-Seq and MR in guiding the diagnosis and therapeutic strategies for Alzheimer’s disease.

## 1. Introduction

Alzheimer’s disease (AD) is a specific, progressive, and irreversible neurodegenerative disorder of the brain, that exhibits several complex pathophysiological mechanisms, predominantly manifesting as aggregation of neurofibrillary tangles and amyloid-β plaques.^[1]^ The principal clinical presentation of AD is the progressive degeneration of cognitive functions, including but not limited to memory, language production decline, and decision-making associative impairment, leading to complete dementia.^[2]^ Epidemiological studies have demonstrated a strong association between AD incidence and age.^[3]^ The incidence is approximately 10% for people aged over 65 years and between 30% and 40% for those aged over 85 years.^[4]^ This rising incidence imposes a substantial socio-economic burden, making AD an increasingly serious public health challenge.^[5]^

AD, a condition marked by bewildering genetic intricacies, suggests a multifactorial genesis.^[6]^ Over the past few decades, extensive studies have identified numerous genetic loci that significantly increase the risk.^[7,8]^ However, the etiological mechanisms of AD are not yet fully understood, and understanding the genetic foundations of AD is crucial for developing targeted interventions and preventive strategies.

Many studies have identified various genetic alterations associated with AD that contribute to the complex pathophysiology of AD,^[9,10]^ highlighting the need for further exploration of the roles these genetic alterations play in different cellular compartments. Genetic alterations in various cellular compartments are vital for understanding their contribution to AD pathology. In this context, our study focuses on the genetic alterations within specific cellular compartments that contribute to AD pathogenesis. We aim to provide insights into the cellular-specific expression patterns of risk genes and their potential roles in the development and progression of AD. By examining these genetic alterations and cellular contributions, we hope to uncover novel therapeutic targets and enhance our understanding of the complex genetic landscape of AD.

In recent years, single-cell RNA sequencing (sc-RNA-Seq) technology has been widely used in the investigation of complex diseases, including AD, revolutionizing our understanding of the cellular mechanisms in AD.^[11,12]^ sc-RNA-Seq can reveal subtle variations in cell heterogeneity and state, providing a robust tool for understanding the cellular and molecular complexities associated with AD.^[13]^ sc-RNA-Seq of brain tissue from patients with AD allows researchers to identify disease specific cell types, states, and molecular markers, thereby revealing key molecular mechanisms in the pathological process.^[14]^

Mendelian randomization (MR) is a sophisticated statistical technique that employs genetic variants as instrumental variables to unearth causal relationships between modifiable intermediate phenotypes and disease outcomes.^[15,16]^ MR offers a new strategy for understanding the genetic background of AD by reducing confounding factors and can help more accurately infer causality in observational studies, thus strongly supporting genetic pathophysiological research in AD.^[17,18]^ In the context of AD, MR provides a valuable tool for validating the functional impact of specific genetic variations identified through sc-RNA-Seq.

This study aimed to integrate high-resolution sc-RNA-Seq with MR to explore the genetic mechanisms associated with AD. This integrated approach offers new insights that are crucial for understanding and potentially mitigating the diseases. The discovery of new biomarkers in this study could pave the way for early diagnosis and targeted treatment strategies, revolutionizing AD management.

## 2. Materials and methods

This study was a secondary analysis that was conducted using publicly available data. Ethical approval was obtained from each original study.

### 2.1. Sequencing data sources and processing

The dorsolateral prefrontal cortex single-nucleus RNA sequencing (sn-RNA-Seq) dataset GSE243292, conducted using the GPL16791 platform, was obtained from the Gene Expression Omnibus (GEO) database (http://www.ncbi.nlm.nih.gov/geo/). Ten samples from GSE243292 were downloaded, including 2 normal human brain tissues without any amyloid or tau pathology, and 8 sample of Alzheimer’s disease presenting both amyloid and tau pathology. These samples were categorized by genetic risk factors for Alzheimer’s disease, such as APOE and TREM2.

The Seurat R software package (version 4.2.0) was used to analyze the sn-RNA-Seq data for further dimensionality reduction, cell clustering, and annotation.^[19]^ First, sn-RNA-Seq data were filtered using the following criteria: each gene was expressed in at least 3 cells. The number of genes expressed per cell ranged from 200 to 4000 (200 < nFeature_RNA < 4000). The mitochondrial content was <5% (percent.mt < 5%), and the number of mRNA in per cell was <12,000 (nCount_RNA < 12,000). The data were then standardized, homogenized, and subjected to harmony analysis to normalize them and remove batch effects. Subsequently, we selected the top 2000 genes with the highest variability in expression to perform a principal component analysis (PCA) and utilized the uniform manifold approximation and projection (UMAP) technology for dimension reduction based on the top 15 principal components. Ultimately, we identified significant marker genes with an adjusted *P* value <.05 and a |log2 (fold change) | >1. Finally, clusters were annotated using known cell markers.

### 2.2. Exposure data

The expression quantitative trait loci (eQTL) data used in this study were downloaded from the eQTLGen Consortium (https://www.eqtlgen.org) database. This eQTL dataset contains information on 10,317 single nucleotide polymorphisms (SNPs) and the expression levels of 19,942 genes from 31,684 participants of European ancestry.^[20]^

### 2.3. Outcome data

Outcome data for Alzheimer’s disease were obtained from the genome-wide association study (GWAS) summary statistics datasets, which were available from the NHGRI-EBI GWAS catalog under accession number GCST90027158 (https://www.ebi.ac.uk/gwas/). This GWAS subset involved 487,511 participants including 39,106 cases, 46,828 proxy cases, and 401,577 controls, all of European ancestry, to identify risk loci associated with Alzheimer’s disease.^[21]^

### 2.4. Mendelian randomization analysis

To begin the Mendelian randomization (MR) analysis, we first searched for instrumental variables (IVs) for the exposure. IVs were defined as SNPs correlated with the exposure data in eQTL and were selected based on a significance threshold for each gene in the whole locus (*P* < 1e^−8^). We calculated linkage disequilibrium (LD) between SNPs, and among SNPs with *R*^2^ < 0.001 (clumping window size = 10,000 kb), only those with *P* < 5e^−8^ were retained as potential IVs. The inverse variance weighting (IVW) method was employed as the primary MR analysis to estimate the causal relationship between SNPs and AD risk. This method possesses the greatest statistical power and is capable of identifying reliable causal estimates in the absence of directional pleiotropy.^[22]^ Furthermore, the weighted median (WM) and MR-Egger methods were employed as complementary approaches to strengthen the findings. The MR-Egger and WM methods were used alongside the IVW estimates because of their ability to deliver reliable estimates under broader conditions, albeit with reduced efficiency. The WM method yields consistent causal estimates when valid IVs contribute to more over 50% of the weight.^[23]^ Conversely, the MR-Egger method identifies multiplicity through its intercept, although this diminishes its statistical power.^[24]^

Additionally, we conducted a leave-one-out sensitivity analysis to assess the impact of individual SNPs on the overall MR estimate.^[25]^ This method identifies and eliminates variants that disproportionately affect the overall estimate by systematically excluding each SNP and recalculating the pooled effect sizes of the remaining SNPs. The removal of each SNP produces a new point estimate and its 95% confidence interval, allowing us to assess the SNPs unique contribution and robustness of the overall results. Estimates after individual SNP removal are summarized, as well as overall estimates when all SNPs are included. By comparing these estimates, we can observe the impact of removing any single SNP on the overall results, thereby determining the robustness of our analysis.

### 2.5. Statistical analysis

The R language (version 4.2.0) was used for statistical analysis. All statistical tests were 2-sided, and *P* < .05 was considered statistically significant.

## 3. Results

### 3.1. Profiling of sn-RNA-Seq and screening of candidate genes

**Figure 1.**
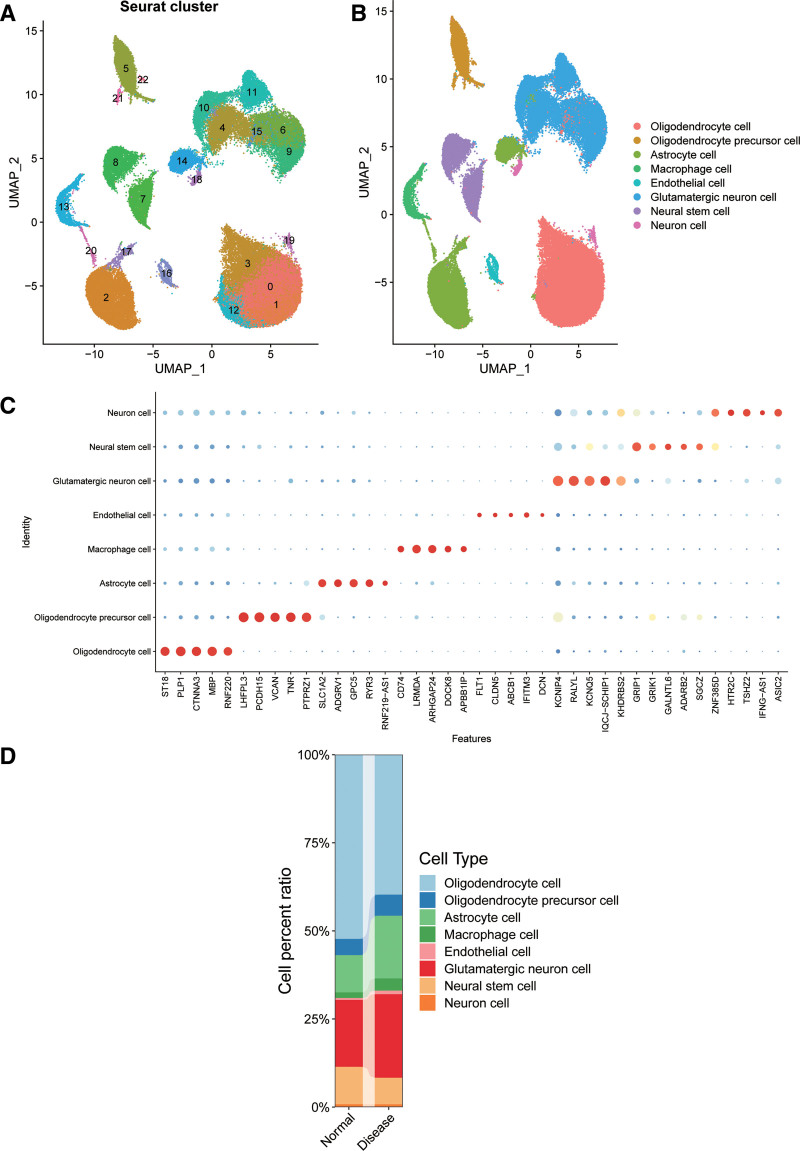
Comprehensive cellular overview of the dorsolateral prefrontal cortex in dataset GSE243292. (A) Twenty-three cell clusters were obtained through UMAP and PCA analysis. (B) Eight cell subgroups were annotated, including oligodendrocytes, oligodendrocyte precursor cells, astrocytes, macrophages, endothelial cells, glutamatergic neurons, neural stem cells, and neurons. (C) Dot plots showing the expression of the marker genes in each cell subgroup. (D) Histograms showing the percentage of each cell subgroup in the disease and control groups.

Through PCA and UMAP dimensionality reduction, 23 cell clusters were identified (Fig. 1A). These 23 cell clusters were annotated into 8 cell subgroups, including oligodendrocytes, oligodendrocyte precursor cells, astrocytes, macrophages, endothelial cells, glutamatergic neurons, neural stem cells and neurons (Fig. 1B), based on the expression of cell-specific markers and significantly enriched genes (Fig. 1C).

When we compared the cellular composition of each cell type between the AD group and the control groups, we found that macrophages were elevated in the AD group compared to the control group (Fig. 1D). The 305 differentially expressed genes in macrophages were selected as the candidate gene sets for our subsequent analysis.

### 3.2. Identification of key genes associated with AD

**Figure 2.**
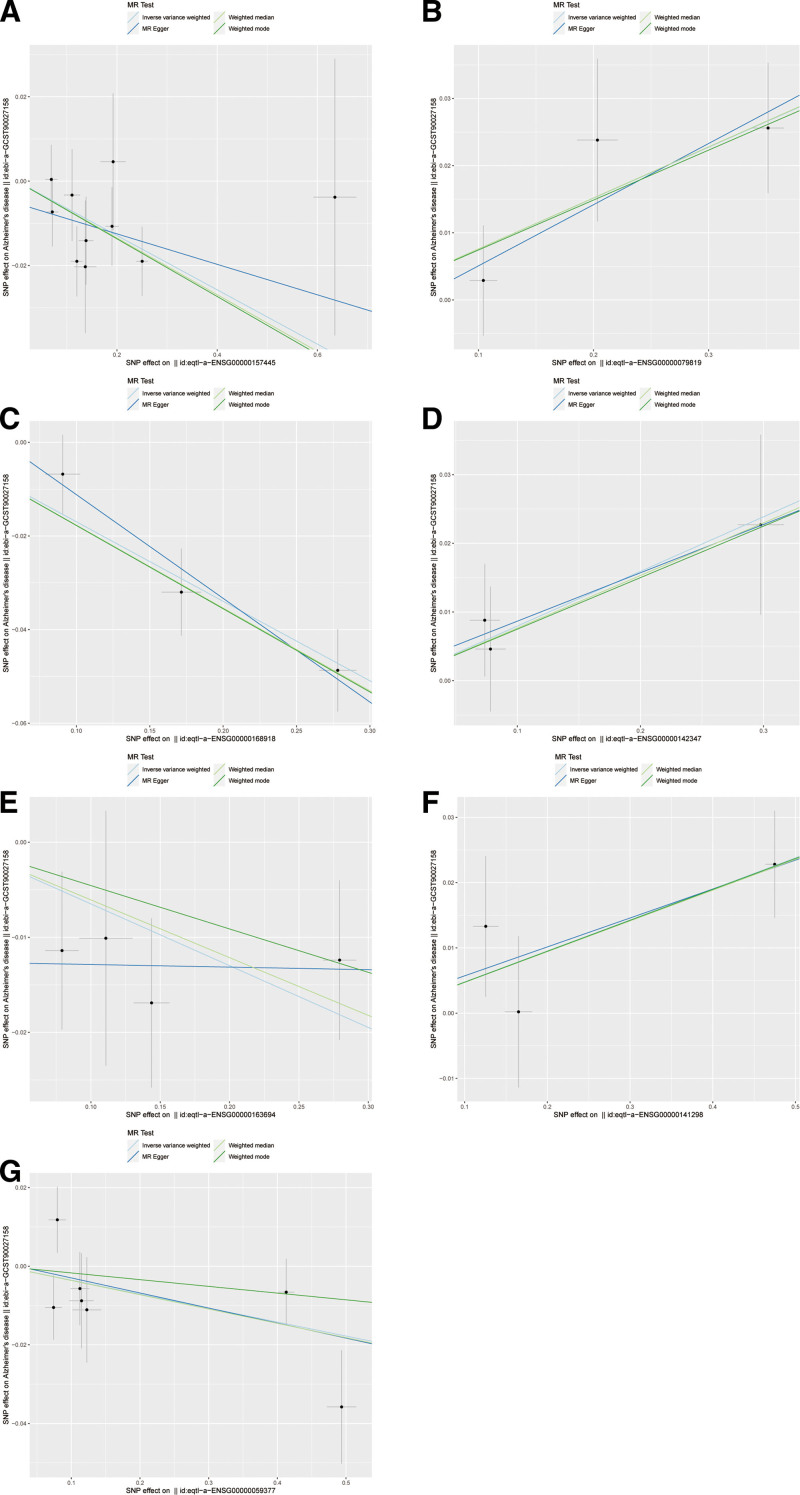
Scatter plot of the causal associations between 7 genes and the risk of AD through MR analyses. Each black dot represents a SNP, plotted from the SNP estimate for 7 key genes and the SNP estimate for AD risk, with a standard error bar. The slope of the each line corresponds to a causal estimate using each of the different MR methods. (A) CACNA2D3 effect on AD risk. (B) EPB41L2 effect on AD risk. (C) INPP5D effect on AD risk. (D) MYO1F effect on AD risk. (E) RBM47 effect on AD risk. (F) SSH2 effect on AD risk. (G) TBXAS1 effect on AD risk. AD = Alzheimer’s disease, MR = Mendelian randomization, SNP = single nucleotide polymorphisms.

**Figure 3.**
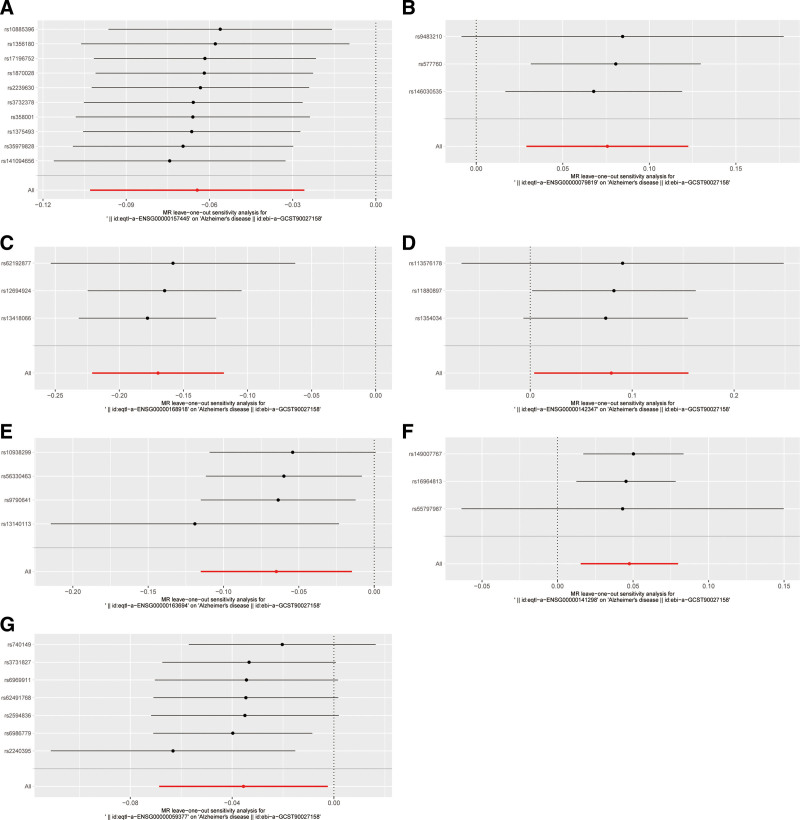
Leave-one-out sensitivity analysis of the association between 7 genes and risk of AD after excluding a particular SNP from the analysis. Each row represents the result after the deletion of the corresponding SNP on the left, and the overall effect is shown at the bottom. (A) CACNA2D3, (B) EPB41L2, (C) INPP5D, (D) MYO1F, (E) RBM47, (F) SSH2, (G) TBXAS1. AD = Alzheimer’s disease, SNP = single-nucleotide polymorphism.

To further identify the key genes affecting Alzheimer’s disease among the 305 differentially expressed genes in macrophages, we used large-scale GWAS summary statistics and publicly available eQTL summary data. Through MR analysis, we obtained the causal relationship of the 7 genes with positive outcomes in the eQTL analysis, using IVW (*P* < .05; Fig. 2). These 7 genes were CACNA2D3, EPB41L2, INPP5D, MYO1F, RBM47, SSH2 and TBXAS1. The presence of the CACNA2D3 (0.938, 0.902 − 0.975, *P* = .001), INPP5D (0.844, 0.802 − 0.888, *P* < .001), RBM47 (0.937, 0.891 − 0.985, *P* = .011) and TBXAS1 (0.965, 0.934 − 0.998, *P* = .036) may be associated with a lower risk of Alzheimer’s disease. In contrast, the gene EPB41L2 (1.079, 1.029 − 1.130, *P* = .001), MYO1F (1.083, 1.004 − 1.168, *P* = .039), and SSH2 (1.049, 1.015 − 1.083, *P* = .004) were associated with a higher risk of developing Alzheimer’s disease (Fig. 2). A sensitivity analysis (leave-one-out) was performed on the causal relationships of these 7 genes to determine their reliability. The results showed that the estimates were similar after deleting each SNP in leave-one-out analysis, indicating that no single SNP had exorbitant impact on the overall estimates. This suggests that the 7 pairs of causal relationships that we selected were robust (Fig. 3).

### 3.3. Expression of key genes in each cell subgroups

**Figure 4.**
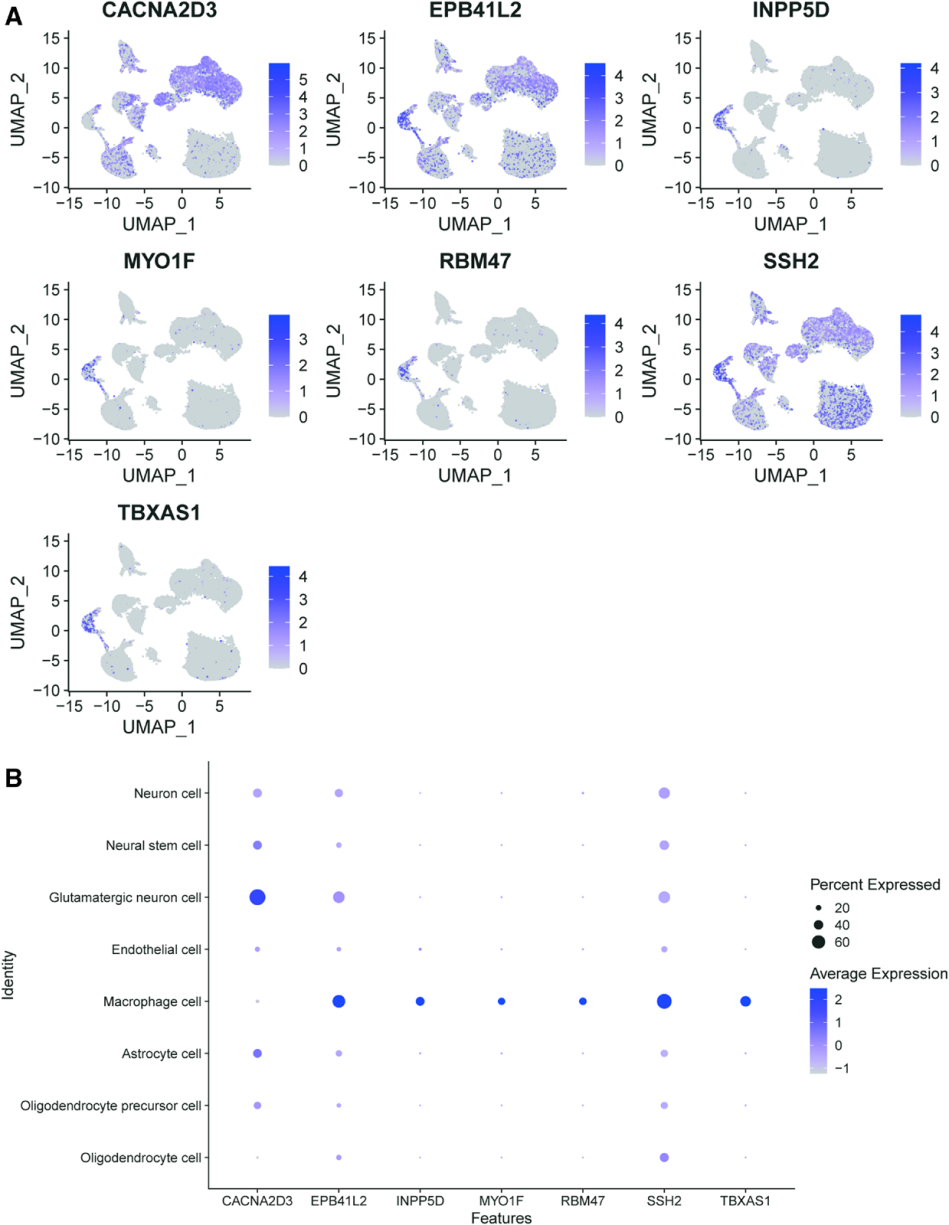
The expression of 7 genes among 8 cell subgroups. (A) Seven genes expression and clustering of 8 cell subgroups. X-axis represents UMAP-1, and Y-axis represents UMAP-2. Each dot represents 1 cell. Color represents level expression of a gene. (B) Dot size showing the percentage of cells expressing the each gene. Larger dot means higher percentage of single cells expressing that gene, and smaller dot means lower percentage of single cells expressing that gene. And dot color reflects the average expression level of each gene in different cell subgroups.

We further analyzed the expression levels of 7 key genes across 8 cell subgroups, including oligodendrocytes, oligodendrocyte precursor cells, astrocytes, macrophages, endothelial cells, glutamatergic neurons, neural stem cells, and neurons (Fig. 4). Overall, the 7 key genes exhibited distinct expression patterns across the 8 cell subgroups (Fig. 4A). The expressions of INPP5D, MYO1F, RBM47, and TBXAS1 were low in oligodendrocytes, oligodendrocyte precursor cells, astrocytes, endothelial cells, glutamatergic neurons, neural stem cells, and neurons, but high in macrophages (Fig. 4B). Significant differences were observed in the expression of CACNA2D3, EPB41L2, and SSH2 across the 8 cell subgroups, both in the percentage of cells expressing these genes and their average expression levels. CACNA2D3 was primarily expressed in glutamatergic neurons, with some expression also observed in oligodendrocyte precursor cells, astrocytes, neural stem cells, and neurons. EPB41L2 and SSH2 were predominantly expressed in macrophages, and moderate expression were also observed in other cell subgroups, such as glutamatergic neurons (Fig. 4B).

## 4. Discussion

This study integrates single-nucleus RNA sequencing (sn-RNA-Seq) with Mendelian randomization (MR) analysis to explore crucial risk genes for Alzheimer’s disease (AD). Utilizing the precision of sn-RNA-Seq analysis, we identified 23 distinct cellular subgroups that were categorized into 8 major cell types. Notably, there was a significant increase in macrophages observed among patients with AD compared to controls. Macrophages play a crucial role in AD pathogenesis, particularly through their interactions with amyloid-β. In the brain, macrophages are activated in the presence of amyloid-β, and these activated macrophages produce neurotoxic substances that contribute to neuronal injury. These substances include reactive oxygen species and inflammatory cytokines such as IL-1β, TNF-α, and IL-6.^[26]^ Thomsen reported significant perivascular macrophage infiltration in the cortex white matter of canine cognitive dysfunction (CCD) animal models, suggesting a role in pathology that might parallel changes observed in early Alzheimer’s disease in humans. Macrophage infiltration correlates with higher amyloid-β levels and the dementia rating score, indicating that more pronounced immune responses are associated with more severe disease.^[27]^ Our findings align with the emerging views that inflammation and immune responses mediated by brain-resident macrophages are pivotal in AD progression. Thus, understanding macrophage activation can help in developing targeted therapies to modulate their activity, potentially slowing disease progression.

Moreover, we identified 7 key genes with significant roles in AD based on the MR analysis. The distinct expression patterns of these key genes across various cellular subgroups shed light on their possible divergent roles in multiple cell types. Among these 7 genes, CACNA2D3, INPP5D, RBM47, and TBXAS1 may be associated with reduced AD risk, suggesting a protective mechanism against the progression of AD. In contrast, genes such as EPB41L2, MYO1F and SSH2 appeared to increase AD risk, thus presenting themselves as potential targets for therapeutic interventions. EPB41L2 (erythrocyte membrane protein band 4.1 like 2), a member of the protein 4.1 family, plays an important role in linking the actin cytoskeleton to the plasma membrane in cells. EPB41L2 is found in specialized subcellular structures in myelinating Schwann cells. As a cytoskeletal protein, EPB41L2 is important for processes such as cell adhesion, migration, and signal transduction. Mutations or dysregulation of EPB41L2 have been implicated in various diseases, including cancer and neurological disorders, reflecting its importance in cell function and integrity.^[28]^ Myo1F is expressed in various immune cells including natural killer (NK) cells, macrophages, dendritic cells, and neutrophils.^[29]^ Myo1F is a critical component of the regulation of macrophage function and inflammation. Myo1F promotes the secretion of pro-inflammatory cytokines such as IL-1β in macrophages, contributing to epithelial damage in inflammatory conditions.^[30]^ Myo1F expression is regulated by the integrin-αVβ3 signaling pathway, which is crucial for the differentiation and polarization of macrophages towards a pro-inflammatory M1 phenotype.^[30]^ In a colitis model, Myo1F deficiency led to reduced secretion of pro-inflammatory cytokines, decreased epithelial damage, improved disease activity, and enhanced tissue repair. In a recent study, researchers identified SHH2 as being significantly associated with AD risk using integrative genomics and blood methylome data analysis.^[31]^ Interestingly, in glutamatergic neurons, we observed an increase in the expression of EPB41L2 and SSH2, 2 genes associated with an increased risk of developing AD. This finding suggests that these genes may play a role in the dysfunction of glutamatergic neurons, which is a key pathological feature of AD.^[32]^ The expression of these genes could potentially contribute to the excitotoxicity and neuroinflammation observed in the disease. Further research is needed to explore the functional implications of these expression changes and their impact on AD pathogenesis.

These findings highlight that disruption of certain genes within macrophages could be central to AD pathogenesis. These findings underscore the critical importance of genetic regulation in immune cells and its potential impact on the development and progression of AD, thereby pointing to new directions for research and therapeutic intervention. Furthermore, the identification of these genes within specific cellular contexts using sn-RNA-Seq emphasizes the role of cellular heterogeneity in AD pathology.^[12,33,34]^ The differential expression of these genes in the disease and control groups underscores the potential mechanistic pathways through which these genes could influence AD progression. Some genes identified in this study were identified in a previous GWAS.^[35]^ Our study extends these results by directly linking them to specific cell types and their states in AD. This not only verifies previous indications of the involvement of these genes in AD but also provides new insights into their specific cellular contributions, which were previously unexplored.

Sensitivity analysis confirmed a causal relationship between these 7 genes and the risk of AD. Our research significantly enhances our understanding of the complexity in AD pathogenesis and opens up new pathways for its early diagnosis and therapeutic intervention. Specifically, the role of macrophages in AD and the expression changes in their related genes provide valuable clues for identifying new therapeutic targets, exploring how modulation of the identified risk genes, especially within macrophages, could influence AD progression opens new avenues for developing targeted therapies.

Overall, through the integration of sn-RNA-Seq and MR, we successfully identified 7 crucial genes associated with AD risk. These findings not only enhance our understanding of AD molecular mechanisms but also lay the foundation for novel diagnostic biomarkers and therapeutic approaches. It is important to note that the generalizability of our findings has certain limitations due to the homogeneity of our study cohort, which consisted exclusively of individuals of European ancestry. This raises concerns about the applicability of our results to other ethnic groups that may have different genetic backgrounds and disease susceptibility factors. Genetic factors can vary significantly among different populations, and thus the identified risk genes and their associations with AD risk may not be consistent across all ethnic groups. Our future research will focus on the functional validation of these key genes identified in the current study with more diverse populations to explore the potential differences in genetic susceptibility to AD. We aimed to delve deeper into understanding the specific roles these genes play in the onset and progression of AD. By pinpointing and validating the functions of key genes, we hope to unlock novel strategies that could potentially alter the course of the disease, offering hope and improved outcomes for patients with AD.

## Acknowledgments

We are grateful to the authors of the all original studies for sharing the data at GEO datasets, eQTLGen Consortium database and GWAS summary statistics in this manuscript.

## Author contributions

**Conceptualization:** Biao Zhang.

**Formal analysis:** Chao Huang.

**Methodology:** Chao Huang, Biao Zhang.

**Resources:** Chao Huang, Fanshu Dai.

**Software:** Ruihao Zhou, Xingya Huang.

**Supervision:** Biao Zhang.

**Visualization:** Ruihao Zhou, Xingya Huang.

**Writing – original draft:** Chao Huang.

**Writing – review & editing:** Fanshu Dai.
